# A novel diabetic foot ulcer diagnostic model: identification and analysis of genes related to glutamine metabolism and immune infiltration

**DOI:** 10.1186/s12864-024-10038-2

**Published:** 2024-01-30

**Authors:** Hongshuo Shi, Xin Yuan, Xiao Yang, Renyan Huang, Weijing Fan, Guobin Liu

**Affiliations:** 1 Department of Peripheral Vascular Surgery, Shuguang Hospital Affiliated to Shanghai University of Traditional Chinese Medicine, Shanghai, China; 2 Guangming Traditional Chinese Medicine Hospital Pudong New Area, Shanghai, China

**Keywords:** Diabetic foot ulcer, Gln metabolism, Molecular clusters, Immune infiltration, Machine learning

## Abstract

**Background:**

Diabetic foot ulcer (DFU) is one of the most common and severe complications of diabetes, with vascular changes, neuropathy, and infections being the primary pathological mechanisms. Glutamine (Gln) metabolism has been found to play a crucial role in diabetes complications. This study aims to identify and validate potential Gln metabolism biomarkers associated with DFU through bioinformatics and machine learning analysis.

**Methods:**

We downloaded two microarray datasets related to DFU patients from the Gene Expression Omnibus (GEO) database, namely GSE134431, GSE68183, and GSE80178. From the GSE134431 dataset, we obtained differentially expressed Gln-metabolism related genes (deGlnMRGs) between DFU and normal controls. We analyzed the correlation between deGlnMRGs and immune cell infiltration status. We also explored the relationship between GlnMRGs molecular clusters and immune cell infiltration status. Notably, WGCNA to identify differentially expressed genes (DEGs) within specific clusters. Additionally, we conducted GSVA to annotate enriched genes. Subsequently, we constructed and screened the best machine learning model. Finally, we validated the predictions' accuracy using a nomogram, calibration curves, decision curve analysis (DCA), and the GSE134431, GSE68183, and GSE80178 dataset.

**Results:**

In both the DFU and normal control groups, we confirmed the presence of deGlnMRGs and an activated immune response. From the GSE134431 dataset, we obtained 20 deGlnMRGs, including CTPS1, NAGS, SLC7A11, GGT1, GCLM, RIMKLA, ARG2, ASL, ASNS, ASNSD1, PPAT, GLS2, GLUD1, MECP2, ASS1, PRODH, CTPS2, ALDH5A1, DGLUCY, and SLC25A12. Furthermore, two clusters were identified in DFU. Immune infiltration analysis indicated the presence of immune heterogeneity in these two clusters. Additionally, we established a Support Vector Machine (SVM) model based on 5 genes (R3HCC1, ZNF562, MFN1, DRAM1, and PTGDS), which exhibited excellent performance on the external validation datasetGSE134431, GSE68183, and GSE80178 (AUC = 0.929).

**Conclusion:**

This study has identified five Gln metabolism genes associated with DFU, revealing potential novel biomarkers and therapeutic targets for DFU. Additionally, the infiltration of immune-inflammatory cells plays a crucial role in the progression of DFU.

**Supplementary Information:**

The online version contains supplementary material available at 10.1186/s12864-024-10038-2.

## Introduction

Diabetic foot ulcer (DFU) is a severe complication of diabetes. Non-healing and persistent inflammation are hallmarks of chronic diabetic ulcers and significant factors contributing to the difficulty of DFU healing. Among approximately 150 million diabetes patients worldwide, 15% to 20% develop foot ulcers, and 40% to 80% of these patients experience ulceration combined with diabetic foot infections [1]. Non-healing DFU generate a significant socioeconomic burden, estimated to cost $4 billion annually, with the cost of each amputation surgery potentially exceeding $53,500 [2]. In the clinical management of DFU, various approaches are used, including intravenous administration of antibiotics and local treatments such as silver ion dressings, insulin topical application, and anti-inflammatory gels. However, long-term use of these treatments can lead to drug resistance and even delay wound healing [3, 4]. Therefore, researching the molecular mechanisms of DFU and developing new therapies that can suppress chronic wound inflammation are crucial for improving treatment outcomes and prognosis for patients with DFU.

Wound healing is a highly complex and regulated biological process. It has been reported that immune-inflammatory regulation plays a crucial role in several stages of chronic wound healing. Neutrophils are the first inflammatory white blood cells to migrate to the wound site, where they eliminate invading pathogens through various mechanisms and initiate subsequent stages of inflammation and non-inflammatory responses [5]. Macrophages play a role in hemostasis, and pro-inflammatory M1 macrophages infiltrate after injury to clear bacteria, dead cells, and debris from the wound site [6]. During the phase of wound healing characterized by proliferation and repair, M2 polarization stimulates the movement and growth of fibroblasts, keratinocytes, and endothelial cells to facilitate the restoration of the dermal and epidermal layers as well as the vascular system. Recent research has highlighted the detrimental effects of lacking lymphocytes in diabetic mice, further deteriorating wound healing. This suggests that when there is a deficiency in innate immune regulatory function, an imbalance in M1 polarization, inadequate angiogenesis, and reduced wound healing can be exacerbated [7].

Glutamine (Gln) is the predominant amino acid found in the blood, and cultured cancer cells efficiently take it up. Researchers have extensively investigated glutamine's functions, including its contribution to aerobic glycolysis, support of the TCA cycle, and provision of reduced carboxylation for lipid synthesis [8]. Furthermore, the metabolism of glutamine is pivotal in enhancing cell growth and viability through its ability to mitigate oxidative stress and preserve the integrity of mitochondrial membranes [9]. Gln serves as an energy source for both tumor cells and immune cells [10]. It is worth noting that M2 macrophages and naive macrophages have different utilization rates of Gln, which affects their inflammatory phenotype. Reduced metabolism of Gln favors pro-inflammatory M1 macrophages [11]. Studies have shown that manipulating glutamine metabolism presents a promising strategy to transition tumor-associated macrophages from an M2 phenotype to an M1 phenotype, thereby bolstering anti-tumor inflammatory immune responses. Moreover, the metabolism of glutamine also exerts an impact on Th1 cell differentiation and the activation of effector T cells, underscoring its potential to reshape the tumor microenvironment and enhance the efficacy of immunotherapy [12]. Although the combination of targeting glutamine along with immunotherapy shows significant promise in oncology, our comprehension of the precise role of glutamine metabolism in immunogenicity and immunotherapy remains constrained. Considering the existing research void, our study seeks to thoroughly examine the interplay between glutamine metabolism and immunotherapy within the framework of DFU. Nonetheless, the exact mechanisms underlying the pathogenesis of DFU remain elusive.

**Fig. 1:**
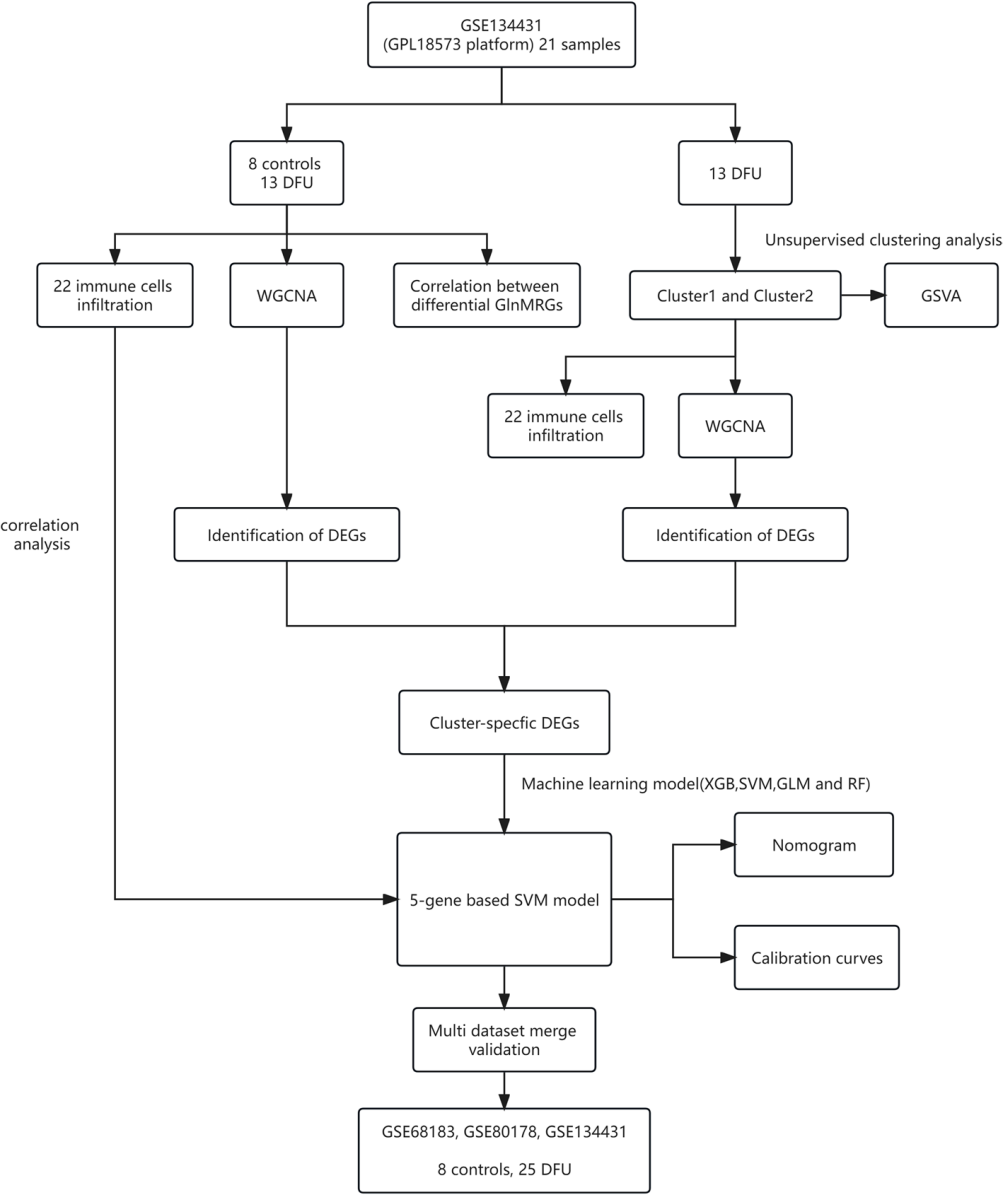
Flow chart of this study

With the development of bioinformatics and machine learning [13, 14], several previous studies have utilized the GEO database to analyze relevant targets of DFU [15, 16]. Hence, we propose that Gln-metabolism related genes (GlnMRGs) play a significant role in DFU development. In this study, unsupervised clustering analysis was used to identify two distinct clusters based on the expression matrix of GlnMRGs. Machine learning models were then constructed using the key modules of DFU and the two clusters of WGCNA [17, 18], and the key models were determined based on the diagnostic sensitivity of the models [19]. This study aims to clarify the potential of GlnMRGs in enhancing immune diagnostics and therapeutic approaches for the management of DFU [20, 21]. The flowchart of this study is illustrated in Fig. 1.

## Materials and methods

### Raw data processing

The analysis utilized the Gene Expression Omnibus (GEO) datasets GSE134431, GSE80178, and GSE68183. GSE134431 was used as the training set, while GSE134431, GSE80178 and GSE68183 served as the validation set. GSE68183 contains samples from 3 DF patients and 3 normal skin samples. GSE80178 contains samples from 9 DF patients and 3 normal skin samples [22]. GSE134431 contains ulcer samples from 13 DF patients and skin samples from 8 DF patients [23]. The dataset of GlnMRGs is obtained from MSigDB. The information for all GEO datasets is presented in Table 1.

**Table 1:** Dataset information

Dataset	Platform	Count	DFU	Control
GSE68183	GPL16686	6	3	3
GSE80178	GPL16686	12	9	3
GSE134431	GPL18573	21	13	8

### Data filtering and processing

Precise mRNA data was acquired by applying Perl-based matching and sorting to the transcript data. Subsequently, for GSE134431, data normalization was performed.

The raw data from the gene expression matrix of the GEO dataset was processed and normalized using the R package “limma” (version 3.52.4). The SVA package was used to correct for batch effects between GSE134431, GSE68183, and GSE80178.

### Cluster analysis of DFU patients

We determined the ideal cluster count by assessing a combination of criteria, including the cumulative distribution function curve, consensus clustering score, and consistency matrix. For this study, we established a maximum cluster count of k = 9.

### Immune cell infiltration

Immune cell composition in DFU tissue was analyzed using CIBERSORT. We used the limma package to construct barplots and corplots to display the results of immune cells. CIBERSORT was used to obtain the deconvolution p-values for each sample through Monte Carlo sampling. A transcriptional feature matrix of 22 immune cells was used for computational simulation. The sum of the percentages of the 22 immune cells in each sample is set to 1. Here, we set the number of computational simulations to 1,000, and samples with a p-value < 0.05 were identified as precise immune cell subtypes.

### Enrichment analysis

We utilized Gene Ontology (GO) and the Kyoto Encyclopedia of Genes and Genomes (KEGG) to investigate biological functions and pathways. We employed R to assess how the differentially expressed GlnMRGs influence biological processes (BP), molecular functions (MF), and cellular components (CC), with this analysis performed using the Gene Set Variation Analysis (GSVA) method. GSVA scores were calculated using the R package “limma” (version 3.52.4), and |t value of GSVA scores| > 2 were considered to be significantly altered.

### Co-expression gene identification

The Weighted Gene Co-Expression Network Analysis (WGCNA) algorithm is used to classify genes and identify relationships between modules and features.. A co-expression network was constructed using the top 25% variable genes from dataset GSE134431. The dynamic tree-cutting method with a threshold of 0.25 was employed to merge modules. Finally, we mapped the modules with the strongest correlation from the two classification methods.

### Building prediction models based on multiple machine learning methods

The identification of cluster-specific GlnMRGs involves the use of WGCNA and the cross-analysis of DEGs within gene clusters. Vnnmap is used for visualizing overlapping genes. We used the “caret” R package to build machine learning models based on two different GlnMRG clusters, including Generalized Linear Model (GLM), Extreme Gradient Boosting (XGB), Support Vector Machine (SVM), and Random Forest (RF). GLM establishes the mathematical expectation of the response variable through a link function and can predict the relationship between linear combinations of variables [24]. XGB can be understood as a parallel prediction model with multiple trees. It iterates continuously, generating a new tree in each iteration, thereby ensuring that the predicted values remain close to the true values [25]. SVM is a type of generalized linear classifier that performs binary data classification through supervised learning. It is unparalleled in addressing small sample sizes, non-linearity, and high-dimensional pattern recognition [26]. In addition, RF is an ensemble machine learning method that uses a variety of independent decision trees to predict classification or regression [27].

Using different clusters as response variables, DEGs specific to selected clusters are chosen as explanatory variables. The DFU samples are randomly divided into training and validation sets in a 7:3 ratio. The “caret” R package automatically adjusts the parameters of these machine learning models through grid search, using default parameters for grid search. Subsequently, evaluation is performed using 5-fold cross-validation. Then, the “DALEX” package (version 2.4.2) is used to interpret four machine learning models and visualize the residual distribution and feature importance. The “pROC” package (version 1.18.0) is used to visualize the area under the receiver operating characteristic curve (AUC). Once the best machine learning model is determined, the top 5 key variables are available for predicting gene correlations in DFU.

### Construction and independent validation analysis of column line chart model

Using the R package “rms” (version 6.3.0), a column line chart model was constructed, with corresponding scores for each predictive variable. The “Total Score” is the sum of the scores for the predictive variables. Additionally, calibration curve and decision curve analysis (DCA) were used to estimate the predictive ability of the column line chart model. The external datasets GSE134431, GSE68183, and GSE80178 were used to validate the model’s ability to differentiate between DFU and normal controls. Furthermore, the R package “pROC” was used to visualize the receiver operating characteristic (ROC) curve.

### Drug-gene interactions

In the realm of disease diagnosis, the progress in bioinformatics has underscored the growing significance of creating biological models and pinpointing efficacious biomarkers. Nonetheless, the utilization of these biomarkers in clinical contexts remains of paramount importance. Predicting drug responses using informative markers is imperative for the future prevention and treatment of DFU. The utilization of the DGIdb database helps predict drug-gene interactions for cross-genetic and generated central genes in the RF model, enabling accurate drug prediction and providing references for therapeutic interventions.

### Ethics approval and consent to participation

This manuscript does not pertain to a clinical trial; therefore, ethical approval and consent for participation are not relevant.

## Results

### Expression of GlnMRGs in DFU patients

**Fig. 2:**
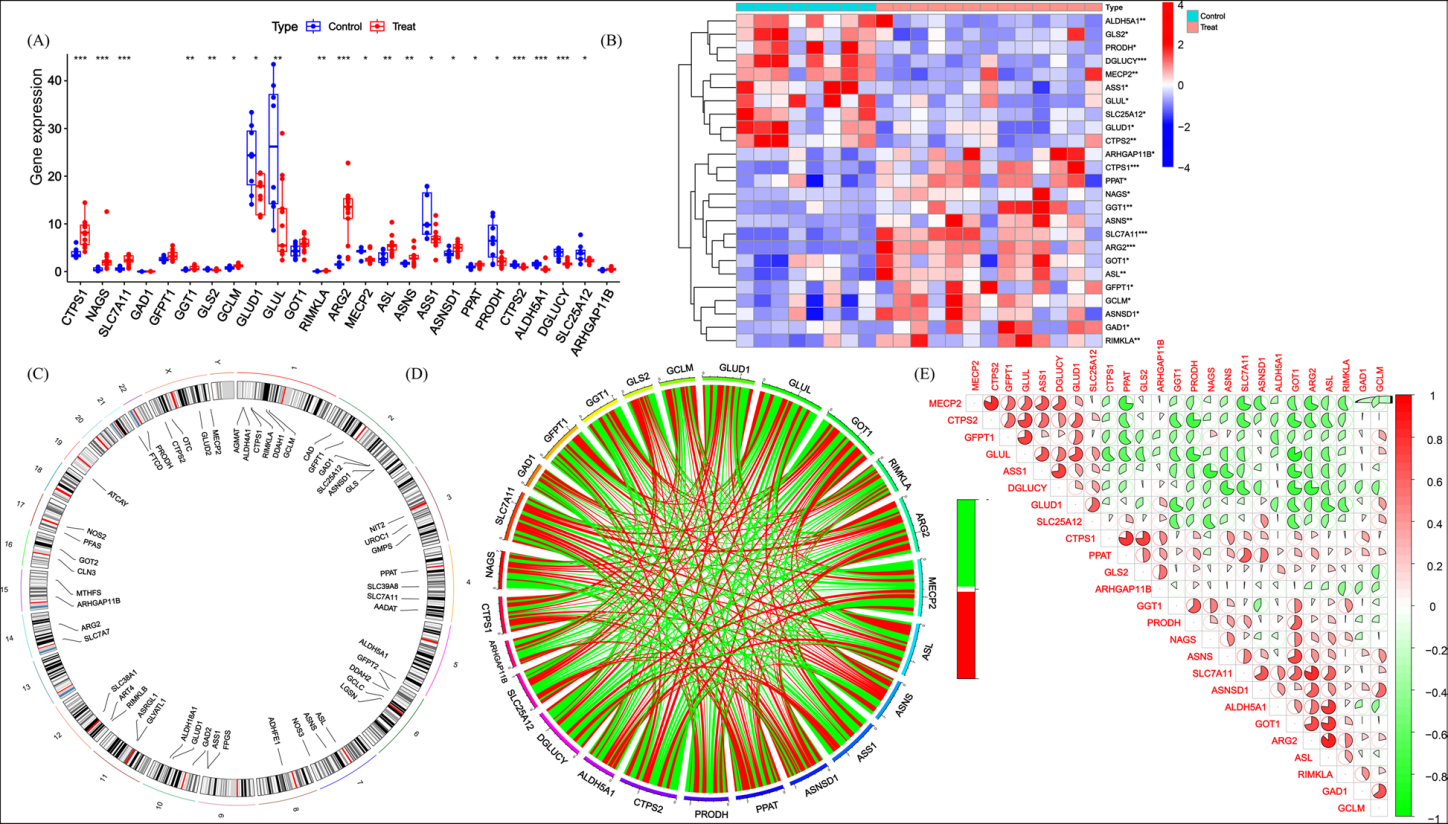
Identification of deGlnMRGs in DFU (**A**) The expression levels of GlnMRGs (**B**) Heatmap of deGlnMRGs (**C**) The location of GlnMRGs on chromosomes (**D**) Gene relationship network diagram of deGlnMRGs (**E**) Correlation analysis of deGlnMRGs. Red and green colors represent positive and negative correlations, respectively. The correlation coefficient was expressed as the area of the pie chart

We identified 20 differentially expressed GlnMRGs (deGlnMRGs). Among them, CTPS1, NAGS, SLC7A11, GGT1, GCLM, RIMKLA, ARG2, ASL, ASNS, ASNSD1, and PPAT showed higher expression levels in DFU testicular tissue. In contrast, the expression level of GLS2, GLUD1, MECP2, ASS1, PRODH, CTPS2, ALDH5A1, DGLUCY, and SLC25A12 in DFU testicular tissue was significantly lower than in normal controls (Fig. 2A, B). The chromosomal positions of the GlnMRGs were calculated and visualized in the form of circles (Fig. 2C). Subsequently, we performed correlation analysis on these genes (Fig. 2D, E). Most of these genes showed positive correlations with each other.

### Immune infiltration analysis

**Fig. 3:**
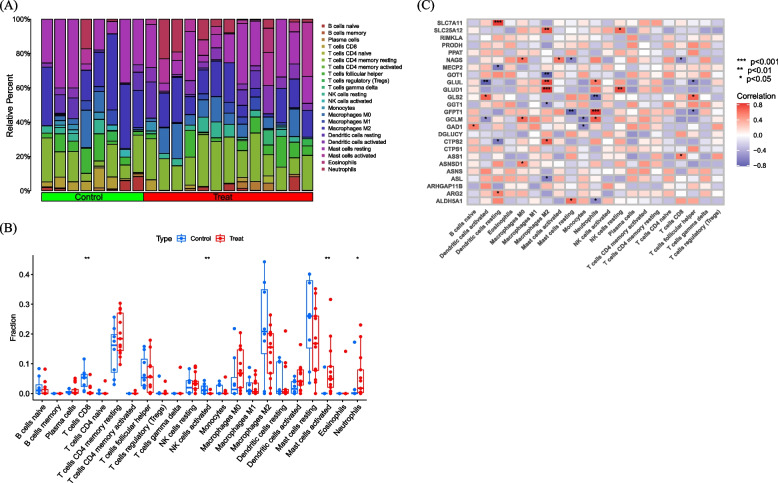
Expression of Immune cells. **A** and **B** Expression of immune cells in different clusters. **C** Correlation between GlnMRGs and immune cells

The immune environment plays a critical role in the occurrence and progression of DFU. The distribution of immune cells in various samples is shown in Fig. 3A. The immune cell differences between the DFU and control groups are depicted in Fig. 3B. Immune cell populations Mast cells activated and Neutrophils are higher in DFU compared to the control group, while population NK cells activated and T cells CD8 are lower in DFU compared to the control group. The correlation analysis between immune cells and GlnMRGs is shown in Fig. 3C.

### Cluster analysis

**Fig. 4:**
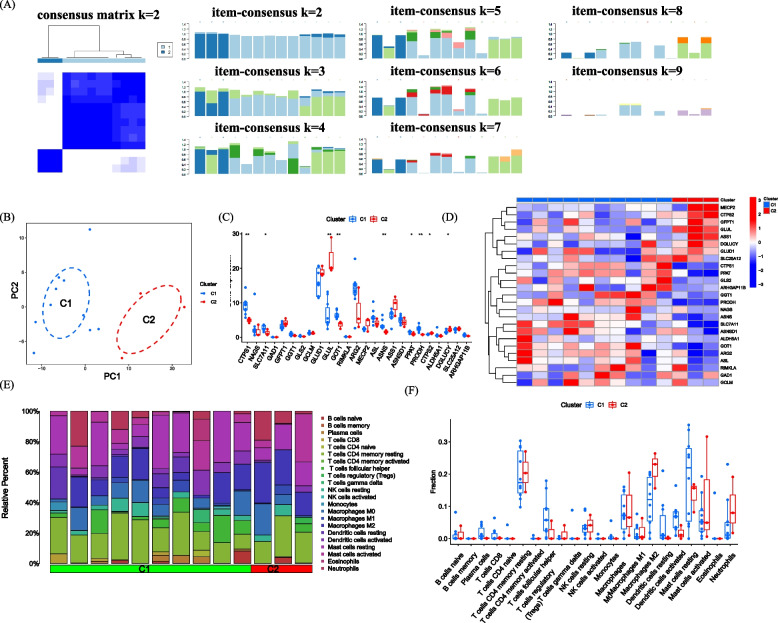
Identification of GlnMRGs clusters in DFU (**A**) Consensus clustering matrix when k = 2. **B** PCA visualized the distribution of the two clusters. **C** Boxplots of GlnMRGs expressed between the two clusters. **D** Heatmap of the expression patterns of the GlnMRGs between the two clusters. **E** Relative abundance maps of 22 infiltrating immune cells between the two clusters. **F** Boxplots of immune infiltration differences between the two clusters

When k is set to 2, the highest within-group correlation is observed, indicating that GlnMRGs can be used to divide DFU patients into two groups (Fig. 4A). Figure 4B demonstrates significant differences in principal component analysis (PCA) between these two clusters. We also examined the expression of GlnMRGs in different clusters based on this cluster analysis. The levels of CTPS1, SLC7A11, GLUL, GOT1, ASNS, PPAT, PRODH, CTPS2, and DGLUCY were found to have significant differences between the two groups (Fig. 4C, D). In addition, we also analyzed the results of immune cell infiltration based on the clusters (Fig. 4E, F).

### Functional enrichment analysis

**Fig. 5:**
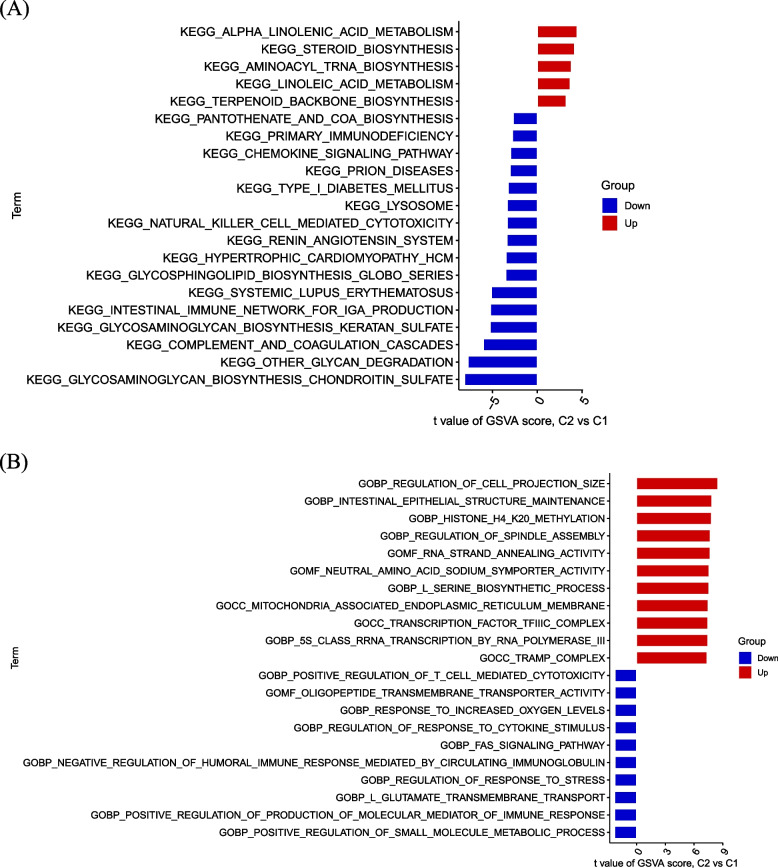
Enrichment analysis for GlnMRGs. **a** KEGG. **b** GO

Performing GSVA enrichment analysis using GlnMRGs. The pathway is mainly enriched in aminoacyl tRNA biosynthesis, steroid biosynthesis, and alpha linolenic acid metabolism (Fig. 5A). The GO analysis results include histone H4 K20 methylation, intestinal epithelial structure maintenance, and regulation of cell projection size (Fig. 5B).

### Gene module selection and construction of co-expression networks

**Fig. 6:**
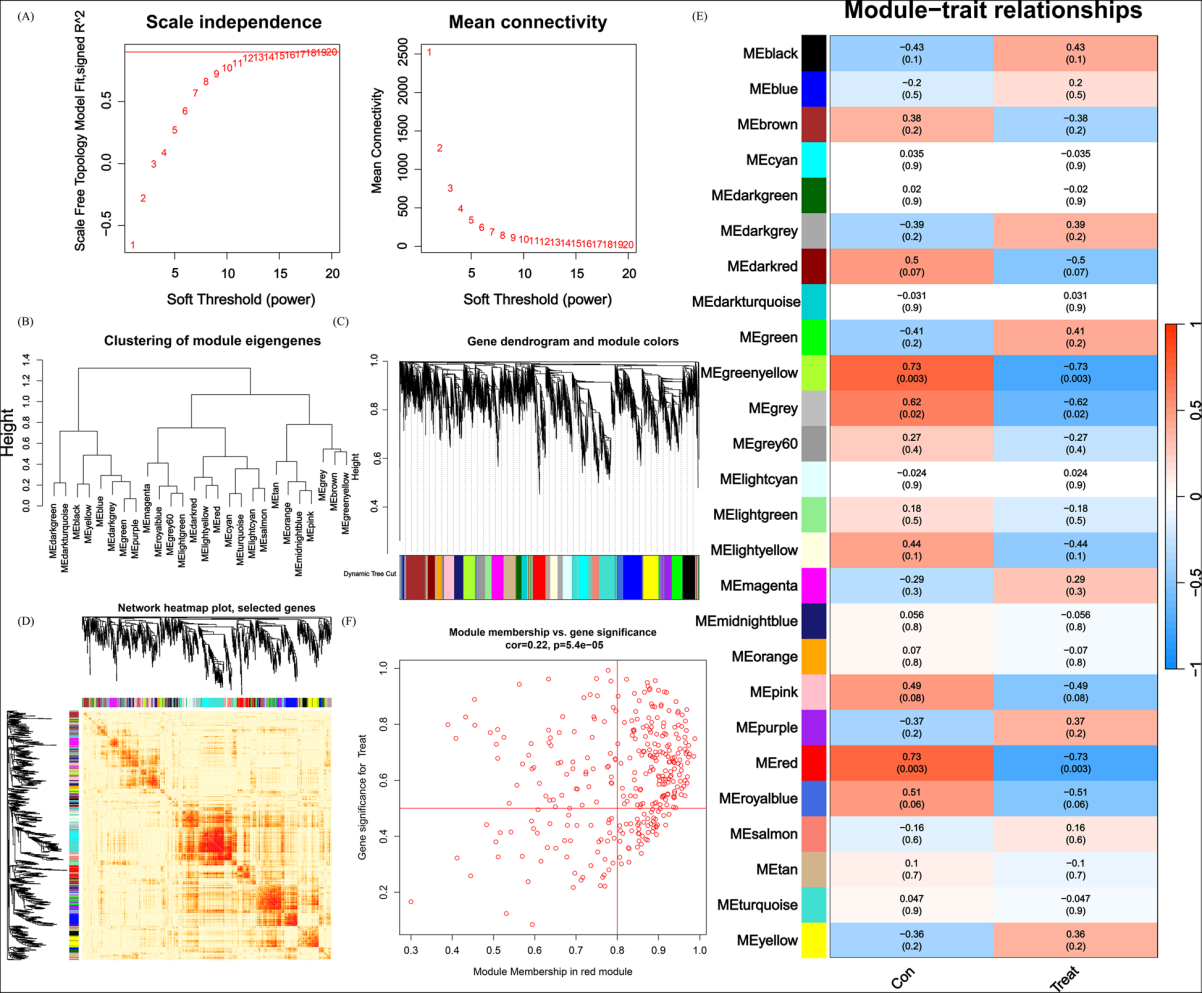
Co-expression network of DEGs in DFU (**A**) Set soft threshold power (**B**) The cluster tree dendrogram of co-expression modules is shown in different colors (**C**) Cluster diagram of module eigengenes (**D**) TOM heatmap of 26 modules (**E**) Heatmap of correlation analysis of module eigengenes with clinical features. Rows and columns represent modules and clinical features, respectively (**F**) Scatter plot of the genetic significance of the blue module members with DFU

We have built a co-expression network for both normal control and DFU patients using WGCNA, and identified key gene modules associated with DFU. We identified the co-expression gene modules under this condition (Fig. 6A). Following that, the dynamic cut algorithm yielded a total of 26 co-expression modules, each represented by distinct colors, and generated a TOM heat map (Fig. 6B,C,D). In addition, by applying the genes from these 26 modules, we analyzed the similarity and continuity of co-expression between module clinical features (normal control and DFU). We found that the red module had the strongest association with DFU, which included 222 hub genes (Fig. 6E). Furthermore, there is a positive correlation between the red module and the module-related genes (Fig. 6F).

**Fig. 7:**
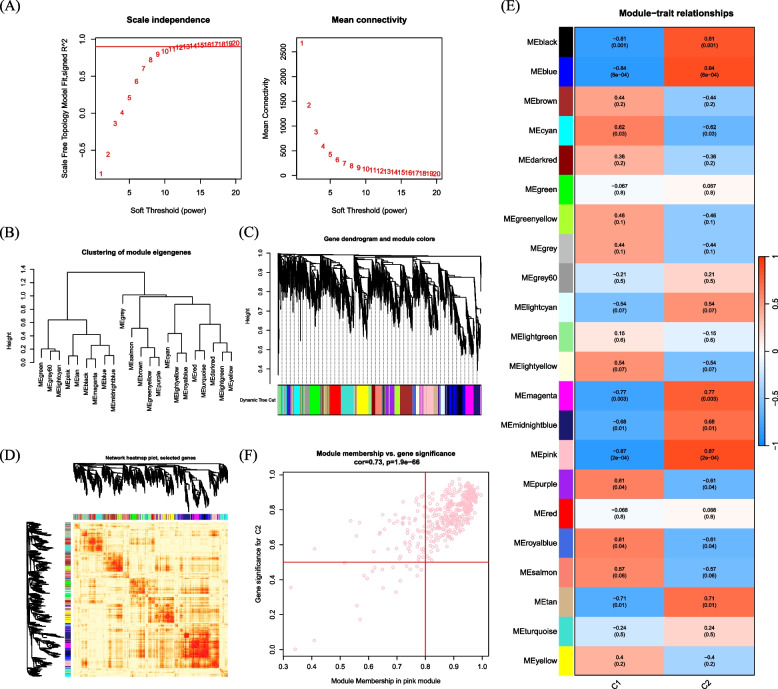
Co-expression network of DEGs between the two Gln clusters (**A**) Set soft threshold power (**B**) The cluster tree dendrogram of co-expression modules is shown in different colors (**C**) Cluster diagram of module eigengenes (**D**) TOM heatmap of 22 modules (**E**) Heatmap of correlation analysis of module eigengenes with clinical features. Rows and columns represent modules and clinical features, respectively (**F**) Scatter plot of the genetic significance of the turquoise module members with Cluster1

Furthermore, we analyzed key gene modules closely associated with Gln metabolism clusters using WGCNA. When the soft threshold parameter β is set to 12 and R^2 is 0.9, a scale-free network is constructed under this condition (Fig. 7A). Twenty-two modules were identified as important. The heatmap displays the TOM of all module-related genes (Fig. 7B,C,D). The examination of the association between modules and clinical characteristics (Cluster1 and Cluster2) underscored the importance of the pink module (Fig. 7E). Furthermore, the correlation analysis results indicate a significant positive correlation between the pink module and its corresponding HUB genes (Fig. 7F).

### Construction of the model

**Fig. 8:**
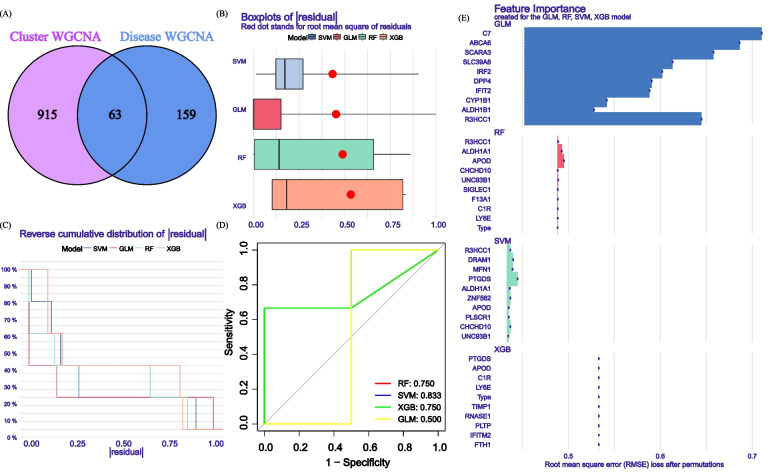
Construction of SVM, RF, XGB, and GLM machine models. **A** Crossover genes of the Gln clusters module and the DFU module. **B** The cumulative residual distribution of the four models. **C** Residual Boxplots of the four machine learning models, where the red dots indicate the root mean square of the residuals (**D**) ROC analysis of four machine learning models with fivefold cross-validation in the test set. **E** The important features in SVM, RF, XGB, and GLM

Mapping the pink module genes of Gln metabolism clusters and the red module genes of DFU, a total of 63 cluster-specific GlnMRGs (Additional file 1: Appendix 1) were obtained (Fig. 8A,B). The residual distribution results of the four models indicate that SVM has the highest residual (Fig. 8B). Figure 8C ranks the top 10 significant feature variables for each model based on root mean square error. The ROC results of the four machine learning models indicate that the AUC value of SVM is 1.000 (Fig. 8D). Therefore, selecting SVM model (R3HCC1, ZNF562, MFN1, DRAM1, and PTGDS) (Fig. 8E) as the best model is because we believe it can best differentiate between different patient groups.

### Evaluating machine learning models

**Fig. 9:**
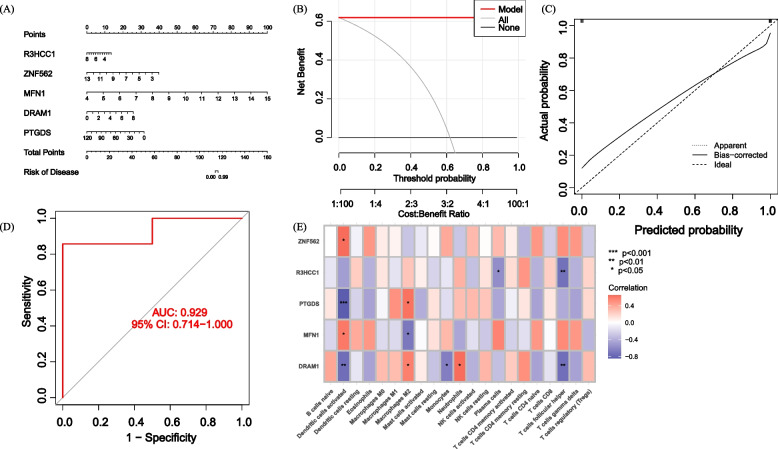
Validation of a 5-gene-based SVM model. **A** Construction of a nomogram to predict DFU risk based on a 5-gene SVM model (**B**, **C**) Calibration curves (**D**) ROC of the 5-gene-based SVM model (**E**) SVM model Model immune-related heatmap

We constructed line plots to evaluate the predictive performance of the SVM model (Fig. 9A). The calibration curve and DCA were used to evaluate the predictive accuracy of the line plot model. The calibration curve shows the minimum error between the actual DFU cluster risk and the predicted risk (Fig. 9B). Furthermore, DCA demonstrates that the line plot has high accuracy and can provide reference for clinical decision-making (Fig. 9C). Subsequently, we used the validation dataset GSE134431, GSE80178, and GSE68183 (Fig. 9D) to validate the model, and the ROC result showed an AUC of 0.929, indicating perfect discrimination. The immune-related analysis of the model genes is presented in Fig. 9E, providing an explanation for the immune function of the model.

### Drug-gene interactions

The all interacting genes were used for drug prediction (Additional file 1: Appendix 2).

## Discussions

DFU represent a complex condition that is unlikely to be effectively addressed using a solitary medication or other intervention measures. The focus of modern drug therapy strategies is to accelerate the healing of chronic wounds and prevent the recurrence of DFU [28]. Over the last three decades, our comprehension of the molecular underpinnings of DFU has notably expanded. This has underscored the potential of biomarkers for the development of diagnostic tools, risk assessment, clinical trials, targeted therapies, and the discovery of novel drug targets [29, 30]. However, the pathogenesis of DFU is still unclear. Therefore, we hypothesize that there is a intricate connection between GlnMRGs and the occurrence of DFU. In our study, we explored our hypothesis through bioinformatics methods to aid in understanding DFU [31]. In this study, we have explored the potential of GlnMRGs as a biomarker in DFU, aiming to contribute to the diagnosis, detection, and treatment of DFU.

Gln metabolism, as an interesting regulatory node, is receiving increasing attention, and it varies in different clinical settings. Gln is among the most plentiful non-essential amino acids found in the body's circulation (amino acids synthesized by the body and not required in the diet), playing a role in nearly all biosynthetic pathways within rapidly dividing cells [32]. Furthermore, it acts as a source of nitrogen for the production of purines and pyrimidines and serves as a precursor for the synthesis of proteins and glutathione. Gln metabolism initiates with the enzyme glutaminase, which deaminates it to yield glutamate. Gln serves as a vital intermediate metabolite with diverse biosynthetic roles within cells [33]. Multiple studies have linked disrupted Gln metabolism to cancer development, and medications aimed at targeting Gln metabolism have received approval for treating various malignant tumors. As cancer advances from premalignant lesions to clinically detectable tumors and subsequently evolves into metastatic malignancies, metabolic requirements and characteristics may undergo alterations. Recent studies have emphasized the role of GlnMRGs in various non-tumor age-related diseases. For example, Wu et al. established metabolic characteristic models for Alzheimer's disease [12] and osteoporosis [34] related to glutamine metabolism. The physiological role of glutamine metabolism in the progression of DFU is not yet clear, and this could be an interesting research direction.

This study utilized unsupervised clustering analysis and further employed the expression profile of GlnMRGs to elucidate the diverse patterns of Gln regulation within GlnMRGs, thereby identifying two distinct clusters of GlnMRGs. In addition, machine learning models were constructed based on disease and key genes identified by WGCNA in the two distinct GlnMRGs clusters, which is an innovative approach in this study. With the progress and development of research, machine learning models are increasingly used for predicting DFU [35]. Compared to traditional univariate analysis, machine learning often employs a multivariate analysis approach that takes into account the relationships between variables. Therefore, machine learning models are more accurate and the results are more reliable. The “caret” R package that we used is a comprehensive machine learning toolkit designed to address prediction problems. Its key feature is the ability to quickly prepare all the necessary components, including data preprocessing, model training, and the entire process of model prediction [36]. We compared the prediction performance of four machine learning models: XGB, SVM, GLM, and RF. The SVM-based prediction model we constructed had very high predictive effectiveness in the test set (AUC = 0.833). In an externally validated dataset, the SVM model based on 5 genes accurately predicted DFU with an AUC of 0.929, demonstrating its good diagnostic value for DFU. Additionally, we constructed a column line chart model for diagnosing DFU using five genes: R3HCC1, ZNF562, MFN1, DRAM1, and PTGDS. Our results showed that the model had good predictive performance, indicating its potential for clinical application. In conclusion, the SVM model based on five genes for distinguishing DFU subtypes was satisfactory.

Using the SVM algorithm, we identified 5 central GlnMRGs (R3HCC1, ZNF562, MFN1, DRAM1, and PTGDS) and validated their diagnostic ability using an external dataset, indicating their potential impact on the pathogenesis of DFU. However, the pathways by which these genes might be linked to the control of particular transcription factors related to Gln management have yet to be elucidated. The R3HCC1 gene encodes a protein consisting of the R3H domain and a coiled-coil region known as coiled-coil containing 1. This protein is thought to exhibit nucleic acid-binding capabilities. Specifically, the R3H domain has the ability to bind to single-stranded DNA and RNA in a sequence-specific manner [37]. As an illustration, the R3H domain modulates the interaction between Rbs1 (a poly[A] mRNA binding protein) and RNA polymerase III [38]. However, it is unfortunate that research on R3HCC1 in DFU remains lacking. Mitochondria are highly dynamic organelles that engage in ongoing fusion and fission processes to preserve their appropriate morphology, a prerequisite for their regular functioning [39]. Mitochondrial fusion entails the combination of two mitochondria and is facilitated by the mitochondrial fusion protein (MFN)-1. MFN1 is situated on the outer mitochondrial membrane and participates in the initial phases of membrane fusion [40, 41]. Research has demonstrated that mice with a deficiency of MFN1 in their skeletal muscles display mitochondrial dysfunction and experience significant muscle atrophy [42]. Simultaneously, mitochondrial dynamics also govern cellular apoptosis [43]. Overexpression of MFN1 promotes mitochondrial fusion and maintains mitochondrial function, while knocking down MFN1 accelerates cellular apoptosis [44]. DRAM1 is a transmembrane protein characterized by six transmembrane domains and is predominantly situated within lysosomes [45, 46]. Being a target gene of p53, DRAM1 assumes a critical role in p53-dependent autophagy and cellular apoptosis [47]. Prostaglandins (PG) are a class of biologically active endogenous metabolites derived from arachidonic acid through the cyclooxygenase (COX) pathway. PTGDS, also known as PGD2, plays a crucial role in vascular function [48]. Research indicates that in diabetic wounds, there is a decrease in COX-1 expression, while COX-2 levels significantly increase [49]. It's important to highlight that a marked enhancement in wound healing is distinguished by a substantial elevation in the synthesis of PGE2/PGD2, which exhibits co-localization with the induced COX-1 within the newly formed tissue surrounding the wound [50].

According to research, DFU wounds exhibit characteristics such as high glucose levels, the accumulation of advanced glycation end-products, hypoxia, and ischemia. There is also a significant presence of mast cells activated, neutrophils, NK cells activated, and T cells CD8 in the local wound microenvironment. Under the complex interplay of numerous cytokines, this ultimately results in an unfavorable immune microenvironment that hinders the rapid repair of DFU wounds [51, 52]. Recently, the immune-inflammatory microenvironment has been identified as one of the reasons for the failure of DFU treatment [53]. The pathological features of an abnormal immune microenvironment in DFU primarily involve changes in the types and functions of immune cells, persistent presence of pro-inflammatory factors leading to sustained tissue damage, and local abnormal cell proliferation. This is responsible for the occurrence of chronic inflammation or acute exacerbations of chronic inflammation at DFU sites, which are fundamentally different from pure acute inflammation. Neutrophils are part of the innate immune system and undergo apoptosis after performing their functions at the wound site. Macrophages eventually phagocytose apoptotic neutrophils, providing a strong signal for the resolution of inflammation [54]. Neutrophils also play a crucial role in vascular formation and maturation. However, in the disrupted immune microenvironment of DFU, coupled with improper extracellular matrix regulation and the pro-inflammatory environment induced by high glucose and hypoxia at the wound site, neutrophils are continuously recruited. Excessive neutrophils hinder vascular formation and maturation, leading to altered vascular permeability and impaired function. This compromised vascular function fails to provide effective microcirculation, weakening wound contraction [55]. The abnormal immune-inflammatory microenvironment leads to impaired NK cell and T cell function, hinders granulation tissue formation, exacerbates inflammation, prevents keratinocytes from migrating and properly epithelializing the wound, delays tissue maturation, and results in the development of chronic non-healing wounds. To successfully treat DFU immune inflammation and restore a normal biological microenvironment at the wound site, both basic and clinical research on DFU must explore unique biomarkers from the perspective of immune-inflammatory interactions.

Research on biomarkers in the context of DFU is still relatively limited. Recently, bioinformatics analysis has become a valuable tool for exploring the intricate and complex connections between cell apoptosis and DFU [56, 57]. A comprehensive study has indicated and identified potential biomarkers associated with DFU through transcriptomics and proteomics bioinformatic models, and a study has shown that CXCL11, DDX60, IFI44, and IFI44L are key hub genes with the potential to serve as molecular targets for immunotherapy in DFU [58]. However, there are currently very few predictive model studies on Gln and DFU. This study, based on the research of Gln mechanisms, provides a reference for identifying effective immunotherapies in the treatment of DFU. Firstly, we collected comprehensive data on GlnMRGs from continuously updated GEO databases to expand upon early research. Secondly, GSE134431 was used as the primary dataset for analysis, and it was combined with GSE80178 and GSE68183 to validate the general pattern in the model. GO and KEGG analyses, as well as GSVA analysis, added credibility to this study. Lastly, there are currently almost no predictive models for GlnMRGs that can provide specific recommendations for future immunoinflammatory research or treatments based on Gln metabolism interference in DFU. This study constructed a diagnostic model for Glu and DFU through machine learning methods, and also combined with immune cell infiltration, enriching our understanding of their immune regulatory interactions. These findings, elucidated through computer algorithms, not only describe the general paradigm of the intricate interactions between Gln, DFU, and the immune system but also pave the way for integrating detailed molecular insights with clinical relevance on a new scientific trajectory. Furthermore, the continuous development of artificial intelligence offers valuable insights for healthcare practitioners and has the potential to shape a more nuanced understanding of DFU and treatment strategies in the future. This can foster rich clinical insights and guide exploration and treatments in the intertwined fields of immunotherapy and foot pathophysiology.

### Translation

Although this study provides a theoretical foundation and research concept, our model still has some limitations. Firstly, the data used in the study is sourced from the GEO database, which poses challenges in determining the quality and reliability of the statistical data. To improve the quality and reliability of the statistical data, we selected GSE134431 as the primary dataset and utilized GSE13443, GSE80178, and GSE68183 for model validation, as these datasets have clear grouping. Secondly, a key challenge is the lack of fundamental understanding of the mRNA-level system related to Gln metabolism and DFU. Therefore, there is a lack of in-depth knowledge about its underlying mechanisms, and future research needs to design foundational experiments for further validation. Lastly, we can further investigate the selection of parameter values in our model, which can be done in future studies in conjunction with basic research to determine the optimal screening parameters for the model.

## Conclusions

The development and progression of DFU result from intricate interactions among diverse targets, signaling pathways, and mechanisms, with regulatory processes that are synergistic and bidirectional. Gln metabolism impacts the synthesis of R3HCC1, ZNF562, MFN1, DRAM1, and PTGDS, and based on this, a diagnostic model was constructed. Future improvements involve increasing the quantity of data sources and conducting more scientific and clinical research to determine whether effective treatments can reduce the immunoinflammatory manifestations in DFU patients by modulating the targets and pathways of Gln metabolism. In conclusion, our research results provide broad potential biomarkers for DFU treatment strategies.

### Supplementary Information

**Additional file 1: Appendix 1.** InterGenes.** Appendix 2.** Drug prediction.

## Data Availability

The datasets used and/or analyzed during the current study are available from the corresponding author on reasonable request.
