# Supplementary material for: A novel diabetic foot ulcer diagnostic model: identification and analysis of genes related to glutamine metabolism and immune infiltration
Source: BMC Genomics. 2024 Jan 30;25:125. doi: 10.1186/s12864-024-10038-2 (PMC10826017; doi:10.1186/s12864-024-10038-2)
Supplement: Supplementary file 1 — Additional file 1: Appendix 1. InterGenes. Appendix 2. Drug prediction. [file 12864_2024_10038_MOESM1_ESM.docx]

**A Novel Diabetic Foot Ulcer Diagnostic Model: Identification and Analysis of Genes Related to Glutamine Metabolism and Immune Infiltration**

**Supplementary appendix to the manuscript**

# Appendix 1

**InterGenes**

| GIMAP5 | CD4 | HLA-A |
| --- | --- | --- |
| R3HCC1 | ALDH1A1 | DAZAP2 |
| ALDH1B1 | PARP14 | LGALS3BP |
| C7 | ZNF562 | F13A1 |
| CYP1B1 | IFI35 | ISG15 |
| IFIT2 | MFN1 | GRINA |
| IRF2 | MX2 | CEBPD |
| SIGLEC1 | SLC39A1 | CD14 |
| TCEAL3 | PROS1 | GLUL |
| UNC93B1 | LGALS9 | C1QC |
| ABCA6 | PDK4 | HLA-B |
| RARRES3 | PLSCR1 | ACTG2 |
| DPP4 | BST2 | PTGDS |
| SCARA3 | PHC2 | PLTP |
| SLC39A8 | PRR13 | RNASE1 |
| SLC15A3 | PLIN2 | LY6E |
| TCN2 | HSPB6 | IFITM2 |
| ENPP2 | LAP3 | TIMP1 |
| CD209 | PIK3R1 | APOD |
| DRAM1 | KRTCAP2 | C1R |
| CD163 | CHCHD10 | FTH1 |

# Appendix 2

**Drug prediction**

| match_term | match_type | gene | drug |
| --- | --- | --- | --- |
| DRAM1 | Definite | DRAM1 | CISPLATIN |
